# The ABC of sealing following left atrial appendage closure

**DOI:** 10.1007/s00392-026-02895-6

**Published:** 2026-04-13

**Authors:** Ali Hamadanchi, Victor K. Eskildsen, Jacob Johnsen, Christian Alcaraz Frederiksen, Bjarke Sihm Stender, Mohamed M. Rahouma Ahmed, Túlio Caldonazo, P. Christian Schulze, Sven Möbius-Winkler, Jesper Møller Jensen, Anders Kramer, Kasper Korsholm, Jens Erik Nielsen-Kudsk

**Affiliations:** 1 Department of Internal Medicine 1, Cardiology, University Hospital of Jena, Jena, Germany; 2 Department of Cardiology, Aarhus University Hospital, Skejby, Palle Juul-Jensens Boulevard 99 (Entrance F), DK-8200 Aarhus, Denmark; 3 Department of Cardiothoracic Surgery, Weill Cornell Medicine, New York, USA; 4 Department of Cardiothoracic Surgery, University Hospital of Jena, Jena, Germany

**Keywords:** Left atrial appendage closure, Intra-device thrombosis, Cardiac computed tomography, Transesophageal echocardiography, Patency, Sealing

## Abstract

**Background:**

Peri-device leak (PDL) following left atrial appendage closure (LAAC) can be diagnosed by both transesophageal echocardiography (TEE) and cardiac computed tomography (CCT). Yet, there is a substantial discrepancy in leak quantification.

**Aims:**

We propose a novel ABC classification of LAA sealing for TEE and CCT based on the pattern of intra-device thrombosis (IDT).

**Methods:**

A single-center observational study including patients undergoing LAAC with Watchman FLX or FLX-Pro between 2022 and 2024. Patients underwent CCT and TEE at 14 days, 45 and 90 days after LAAC. Images were analyzed by three blinded investigators. LAA sealing was classified by four distinct morphological patterns of IDT: Type A: Complete IDT (> 95% of the device). Type B: Incomplete IDT (50–95% of the device). Type B1: Incomplete thrombosis of the distal parts of the device. Type B2: Incomplete thrombosis in the proximal part near the atrial device surface. Type C: Partial IDT (< 50% of the device).

**Results:**

Overall, 68 patients (mean age 76.1 years, 37% female) with complete CCT follow-up were included. At 90-day IDT classifications by CCT were Type A (53.8%), Type B1 (29.2%), Type B2 (0%), and Type C (16.9%). Type B2 was present on 14 days (1.5%) and 45 days (3.1%). PDL was observed in only 2.9% (95% CI, 0.7%–14.5%) of Type A, 73.7% (95% CI, 52.4%–93.6%) of Type B1, and 100% of Type C. Inter- and intra-reader agreements were very high for CCT and substantial for TEE imaging.

**Conclusion:**

Type A was associated with sealing irrespective of imaging modality, while Type C was always an indicator of PDL. This unifying classification was highly reproducible across imaging modalities and among readers of differing levels of experience.

**Graphical Abstract:**

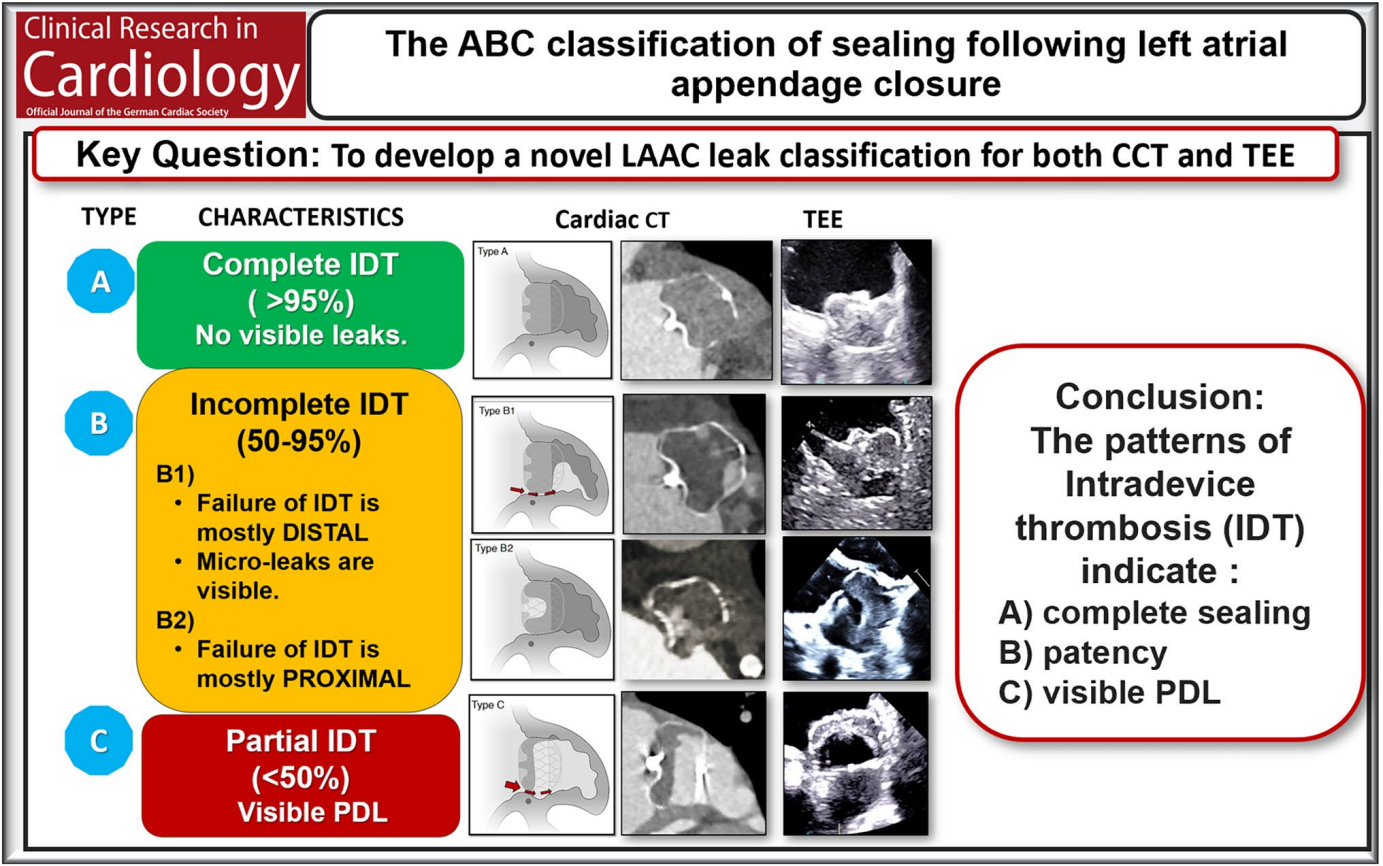

**Supplementary Information:**

The online version contains supplementary material available at 10.1007/s00392-026-02895-6.

## Introduction

As the prevalence of non-valvular atrial fibrillation (AF) rises, the demand for transcatheter left atrial appendage closure (LAAC) procedures may increase substantially [1–3]. This procedure has become an effective non-pharmacological approach for stroke prevention in selected patients with AF [4–9]. LAAC aims at complete sealing of the left atrial appendage [10]. Despite growing operator experience and improved device designs, peri-device leak (PDL) remains considerable in clinical practice [11, 12]. This could be partially explained by the controversial definition of significant versus non-significant PDL, arbitrarily defined by sizing limits of 5 or 3 mm for the diameter of the peri-device color Doppler jet [13].

TEE is currently the mainstay modality for device surveillance [14]. Nevertheless, cardiac computed tomography (CCT) has become a well-established modality due to the advantages of non-invasive acquisition of isotropic high-resolution images, with a higher sensitivity to detect PDL compared to TEE [8, 15, 16]. Observational evidence indicates a significant discrepancy between PDL detected by either TEE or CCT in terms of clinical relevance [11, 17]. A meta-analysis of more than 61,000 patients with AF who underwent LAAC revealed that only PDL detected by TEE was associated with adverse events, primarily thromboembolism [11]. Another recent study of 519 patients with CCT surveillance at 90 days post LAAC, on the other hand, concluded that even CCT-detected LAA patency with or without visible leak, was also associated with increased risk of stroke/transient ischemic attack [17].

In addition, a meta-analysis of 17 studies (2036 LAAO patients, mean follow-up 1.5 years) found that LAA patency on CCT nearly doubled thromboembolism risk (OR 1.87), while PDL showed a similar but nonsignificant trend (OR 1.50). Bayesian analyses produced comparable results. LAA patency was also linked to higher all-cause mortality (OR 2.44) [2].

Therefore, the available evidence supports an association between LAA patency and thromboembolic risk.

It remains however unclear whether the TEE sizing thresholds for PDL can be extrapolated to CCT [7].

The contentious nature of these findings necessitates a shared vocabulary among modalities. Recently, a non-Doppler-based echocardiographic evaluation of PDL based on intra-device thrombosis (IDT) patterns has been introduced [18]. This could potentially improve the association with CCT findings; however, the methodology lacks validation. Here, we propose a novel, easy-to-implement and unifying CCT-TEE classification of IDT patterns, intending to evaluate device sealing following LAAC.

## Methods

### Study design and population

This was a single-center observational cohort study of consecutive patients undergoing LAAC with Watchman FLX™ (WF) or Watchman FLX-Pro™ (WFP); (Boston Scientific, Marlborough, USA) between 2022 and 2024 at Aarhus University Hospital, Denmark as part of the FLX-CT studies (NCT05567172, NCT05324371). The study conformed to the Declaration of Helsinki and was approved by the Danish National Ethics Committee (2,207,880 and 2,215,247).

All implantations were performed in local anesthesia guided by intracardiac echocardiography using the Watchman TruSeal access system. Single antiplatelet therapy (ASA 75 mg daily) was given for at least 6 months after implantation.

Preprocedural planning and the LAAC procedure were previously described in detail [19].

Patients participating in these trials were scheduled for 14-, 45-, and 90-day follow-up including both CCT and TEE. Cardiac CT was omitted in patients with a glomerular filtration rate ≤ 30 mL/min. Patients with both CCT and TEE available at 90 days were included in the present analysis. An experienced reader and a novice reader (20 training cases) analyzed both CCT and TEE images. All imaging analyses were performed with readers blinded to all clinical data and outcomes.

### Definitions of the ABC classification

The ABC-classification was developed to be applicable in both CCT and TEE based on observations of four distinct morphological patterns of IDT:

- Type A: Complete IDT covering > 95% of device volume
- Type B: Incomplete IDT (50–95% of device volume)
  o Type B1: Incomplete thrombosis of the distal parts of the device.
  o Type B2: Incomplete thrombosis in the proximal part near the atrial device surface.
- Type C: Partial IDT. Thrombosis of < 50% of the device volume.

### TEE imaging

**Fig. 1:**
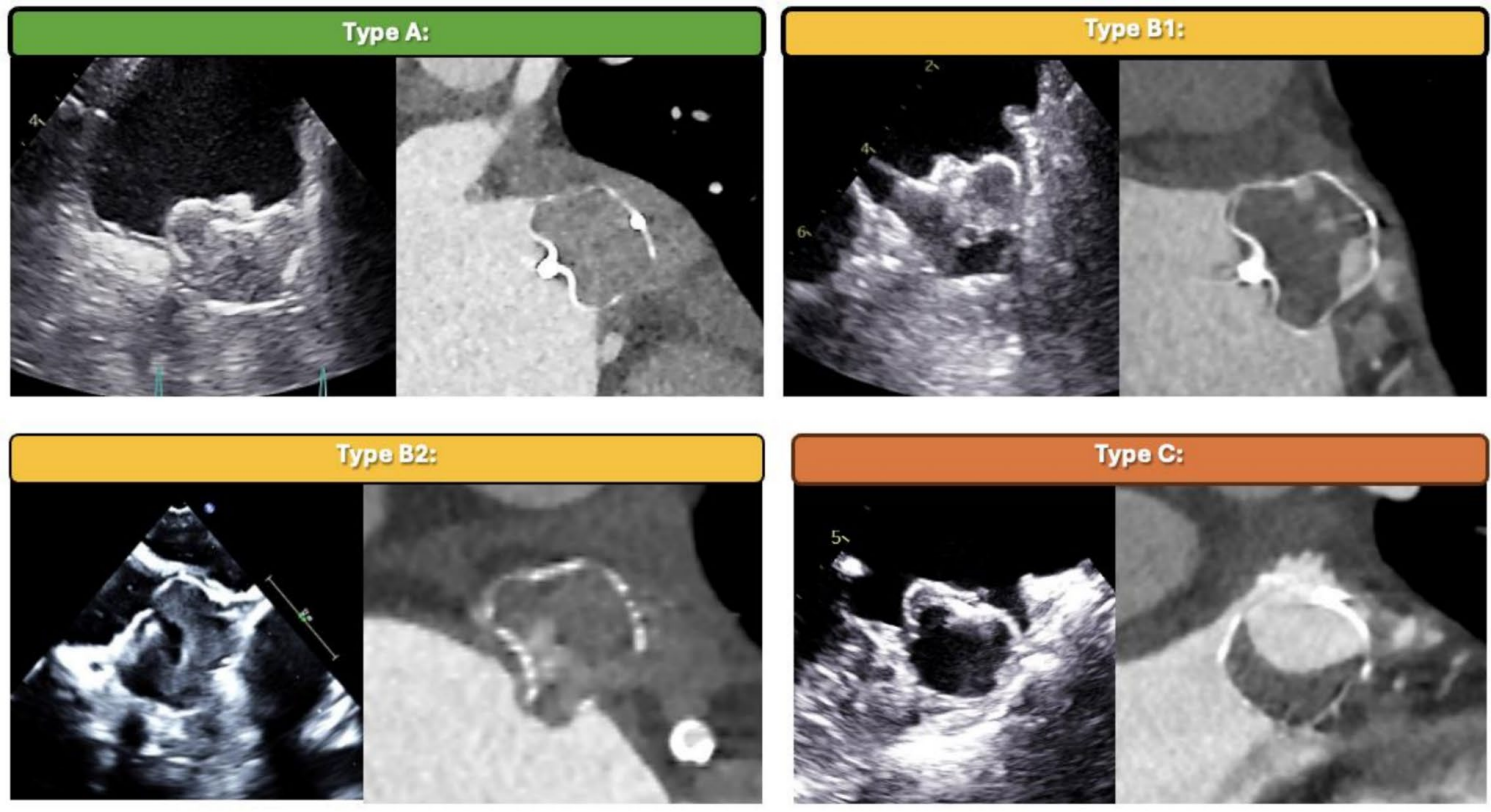
The ABC-classification of IDT following LAAC. Prototypical images of classes A, B1, B2, and C in both TEE and CCT side by side

TEE follow-up was performed using the GE 6VT-D probe with the Vivid E95 machine (GE Healthcare, Chicago, Illinois). The implanted device was identified and visualized in multiple planes with focused, systematic evaluation for PDL at approximately 15° intervals ranging from 0 to 150°. Assessment of PDL was performed using color Doppler with the Nyquist scale set between 20 and 50 cm/s [15]. The width of the color Doppler jet was measured in multiple views, and the largest measurement was recorded (Fig. 1). The PDL was quantified according to the width of the color Doppler jet into < 1 mm, 1–3 mm, or > 3 mm.

### Cardiac CT imaging

The detailed acquisition protocol has previously been described [20, 21]. In short, all CCT scans were performed using the Siemens SOMATOM Force (Siemens Healthcare CT Systems, Forchheim, Germany) using electrocardiographic gating and high-pitch spiral acquisition at *t* = 0 s (Flash 1 [F1]) and *t* = 4 s (Flash 2 [F2]).

### Statistical analysis

The diagnostic performance of TEE and CT imaging across the 14 -,45- and 90-day timepoints was assessed by sensitivity, specificity, and accuracy of TEE-imaging in classifying IDT in accordance with CT results. To assess overall sensitivity and specificity across the IDT types presented in the ABC classification, mean sensitivity and specificity were calculated with CT as the reference. Diagnostic consistency and variation between TEE, CT (F1), and CT (F2) were visualized by flow diagrams (Sankey plots), while receiver operating characteristic (ROC) curves were used to evaluate the diagnostic accuracy for the IDT categories (A, B, and C). The area under the curve (AUC) was used to assess the test’s discriminatory ability across modalities.

Inter- and intra-reader agreement for both CCT and TEE was assessed by absolute agreement, unweighted Cohen’s kappa, while reliability was measured through Kendall’s Tau $-b$. Two rounds of CCT imaging analysis from both investigators were performed with inter- and intra-reader statistics calculated for early and late CCT acquisitions. For TEE analysis, one round of both inter- and intra-reader analysis was performed.

The prevalence of PDL by IDT-class was reported and the association was tested by Fisher’s exact test. A *p*-value < 0.05 was considered statistically significant.

Statistical analyses of diagnostic performance were performed using the table one, caret, and pROC packages in R version 4.4.1 within RStudio. Descriptive statistics on demographic and baseline characteristics, as well as inter-/intra-reader analyses and Fisher’s exact tests, were performed using STATA version 17 (STATA IC, StataCorp, College Station, Texas).

## Results

### Patient population

A total of 68 patients underwent LAAC with either the Watchman FLX™ (25 patients, 36.8%) or the Watchman FLX-Pro™ device (43 patients, 63.2%) between November 2022 and June 2024 as part of the FLX-CT studies (NCT05567172, NCT05324371).

CCT follow-up was available in 67 patients (98.5%) at 14 days, 64 (94.1%) at 45 days, and 65 (95.6%) at 90 days. The proportion of patients with available CCT imaging at all 3 timepoints was 62/68 (91.2%). For TEE, this proportion was 76.5%, with TEE imaging available in 62 (91.2%), 59 (86.8%), and 54 (79.4%) patients at 14, 45, and 90 days, respectively. There were more females with IDT patterns classified as Types B1 and C at 90 days. Baseline characteristics are summarized in Table 1.

**Table 1:** Baseline characteristics and type of intra-device thrombosis by CCT

**Type of intra-device thrombosis at 90 days**						
			A	B1	C	Total
			*N* = 35 (53.8%)	*N* = 19 (29.2%)	*N* = 11 (16.9%)	*N* = 65 (100%)
	No. of patients	(%)				
Gender						
	Female		9 (25.7)	9 (47.4)	6 (54.5)	24 (36.9)
Age at LAAC			76.13 (8.10)	77.95 (5.49)	72.81 (7.15)	76.01 (7.36)
BMI			26.13 (4.61)	25.32 (4.41)	29.56 (4.62)	26.50 (4.71)
Type of atrial fibrillation						
	Paroxysmal (<7 days, terminates spontaneously)		18 (51.4)	11 (57.9)	7 (63.6)	36 (55.4)
	Persistent (≥7 days)		4 (11.4)	0 (0.0)	1 (9.1)	5 (7.7)
	Permanent (>7 days, no rhythm control)		11 (31.4)	8 (42.1)	2 (18.2)	21 (32.3)
	Unknown		2 (5.7)	0 (0.0)	1 (9.1)	3 (4.6)
CHA_2_DS_2_-VASc			3.77 (1.29)	4.32 (1.80)	4.46 (1.75)	4.04 (1.54)
HAS-BLED score			2.49 (1.04)	2.74 (0.81)	2.27 (0.65)	2.52 (0.92)
Baseline eGFR			72.94 (13.58)	71.58 (17.44)	76.36 (14.60)	73.12 (14.81)
	eGRF≤45 ml/min/1.73 m^2^		1 (2.9)	2 (10.5)	0 (0.0)	3 (4.6)
Left ventricular ejection fraction			56.25 (6.48)	54.1 (5.90)	58.70 (2.83)	56.02 (5.97)
	LVEF≤45		2 (5.7)	3 (15.8)	0 (0.0)	5 (7.7)
Valvular heart disease			4 (11.4)	2 (10.5)	0 (0.0)	6 (9.2)
Implanted device						
	Watchman FLX		13 (37.1)	10 (52.6)	0 (0.0)	23 (35.4)
	Watchman FLX Pro		22 (62.9)	9 (47.4)	11 (100.0)	42 (64.6)

*LAAC* left atrial appendage closure, *BMI* body mass index (kg/m^2^), *eGFR* estimated glomerular filtration reader, *LVEF* left ventricular ejection fraction. Numbers shown are mean (SD) for continuous variables and (%) for categorical variables

**Fig. 2:**
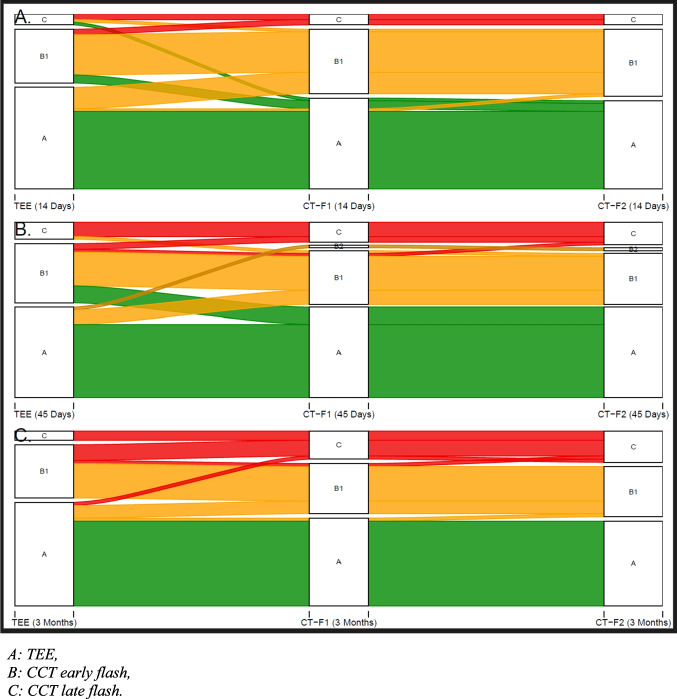
Sankey plot of temporal development of IDT-types ( **A**, **B1**, **B2** and **C**)  prevalence from 14 through 45 to 90 days

At 90-day CCT follow-up, IDT patterns were distributed as follows: 35/65 (53.8%) Type A, 19/65 (29.2%) Type B1, 0/65 (0%) Type B2, and 11/65 (16.9%) Type C. Instances of Type B2 were present at 14 days (1.5%) and 45 days (3.1%). Figure 2 and Table 2 display the temporal development of IDT-type. Three cases of device-related thrombus (DRT) were found at 90 days corresponding to a DRT rate of 4.6%.

**Table 2:** Prevalence of IDT type at 14, 45, and 90 days by imaging modality

	14 days		45 days		90 days	
	CCT (***N*** = 67)	**TEE (*N* = 62)**	CCT (***N*** = 64)	TEE (***N*** = 59)	CCT (***N*** = 65)	TEE (***N*** = 54)
		No. of patients (%)				
Type A	37 (55.2)	38 (61.3)	34 (53.1)	32 (54.2)	36 (55.4)	34 (62.9)
Type B1	25 (37.3)	20 (32.3)	20 (31.3)	21 (35.6)	18 (27.7)	17 (31.5)
Type B2	1 (1.5)	0 (0.0)	2 (3.1)	0 (0.0)	0 (0.0)	0 (0.0)
Type C	4 (6.0)	4 (6.5)	8 (12.5)	6 (10.2)	11 (16.9)	3 (5.6)

*CCT* cardiac computed tomography, *TEE* transesophageal echocardiography

Variables shown as *N* (%)

### Association between pattern of IDT and PDL

When analyzed by CCT, PDL at 90 days was observed in only 2.9% with Type A IDT and 73.7% with Type B1, while both PDL and distal LAA patency were observed in all instances of Type C IDT (Table 3). Fisher’s exact test was employed to test the association between IDT category and presence of PDL yielding *p* < 0.001. The same association was also tested for TEE-detected PDL and yielded *p* < 0.001. Despite a small sample size, we observed a tendency towards larger PDL sizes in patients with Type C at 90 days compared with Type B1. The mean width of color Doppler jets in TEE-detected PDL was 3.33 mm (95% CI, −0.46–7.13 mm) in Type C and 1.23 (95% CI, 0.56–1.98 mm) in Type B1 (*p* = 0.154).

**Table 3:** Readers of PDL by type of intra-device thrombosis and imaging modality at 90 days

Grade of intra-device at thrombosis at 3 months					
		A	B1	C	Total
		*N* = 35 (53.8)	*N* = 19 (29.2)	*N* = 11 (16.9)	*N* = 65 (100)
CCT					
	Any PDL detected	1 (2.9)	14 (73.7)	11 (100)	26 (40)
Grade of intra-device at thrombosis at 3 months					
		A	B1	C	Total
		*N* = 34 (53.8)	*N* = 17 (29.2)	*N* = 3 (16.9)	*N* = 54 (100)
TEE					
	Any PDL detected	0 (0)	11 (64.7)	3 (100)	14 (25.9)
	PDL size < 1 mm	-	4 (23.5)	0	4 (7.4)
	PDL size = 1–3 mm	-	6 (35.3)	1 (33.3)	7 (13.0)
	PDL size > 3 mm	-	1 (5.88)	2 (66.7)	3 (5.6)
	Mean PDL size	-	1.3 (1.1)	3.3 (1.5)	1.7 (1.4)

*PDL* peri-device leakage, *CCT* cardiac computed tomography, *TEE* transesophageal echocardiography

All categorical variables are shown as *N* (%). Continuous variables are shown as mean (SD)

### Inter- and intra-reader reproducibility

The inter-reader reproducibility of the ABC-classification in CCT imaging was substantial to almost perfect as assessed by Cohen’s unweighted kappa as shown in Table 4. Likewise, Tau-b correlations were high, indicating strong reliability (see Supplementary Tables 1–2) for grading of reproducibility. Intra-reader reliability was also substantial to almost perfect (see Supplementary Table 3).

**Table 4:** Inter-reader reproducibility of ABC-classification on cardiac CT at 14, 45, and 90 days on Flash 1 (0 s) and Flash 2 (4 s)

**CT inter-reader reproducibility (Round 1)**					**CT inter-reader reproducibility (Round 2)**				
***Time and flash***	***Agreement***		***Reliability***		***Time and flash***	***Agreement***		***Reliability***	
14 days F1	Agreement:	96.61% (42.55%)	Tau-*b*:	0.95	14 days F1	Agreement	95.45% (44.65%)	Tau-*b*:	0.93
	*Kappa:*	0.94				*Kappa*	0.92		
14 days F2	Agreement:	98.04% (48.10%)	Tau-*b*:	0.96	14 days F2	Agreement	95.45% (44.74%)	Tau-*b*:	0.92
	*Kappa:*	0.96				*Kappa*	0.92		
45 days F1	Agreement:	92.73% (37.92%)	Tau-*b*:	0.91	45 days F1	Agreement	95.24% (40.31%)	Tau-*b*:	0.93
	*Kappa:*	0.88				*Kappa*	0.92		
45 days F2	Agreement:	92.73% (35.90%)	Tau-*b*:	0.91	45 days F2	Agreement	93.65% (37.97%)	Tau-*b*:	0.92
	*Kappa:*	0.89				*Kappa*	0.90		
90 days F1	Agreement:	87.27% (38.38%)	Tau-*b*:	0.79	90 days F1	Agreement	89.06% (41.04%)	Tau-*b*:	0.80
	*Kappa:*	0.79				*Kappa*	0.81		
90 days F2	Agreement:	87.50% (37.02%)	Tau-*b*:	0.83	90 days F2	Agreement	90.62% (40.70%)	Tau-*b*:	0.85
	*Kappa:*	0.80				*Kappa*	0.84		

*Agreement*, absolute agreement (expected chance agreement); *Kappa*, Cohen’s unweighted kappa statistic. *Tau-*b: Kendall’s tau

Inter-reader reproducibility of the classification system applied to TEE imaging was moderate to substantial while intra-reader reliability was substantial to almost perfect as seen in Table 5. Estimates of Kendall’s tau-*b* also reflected overall good reliability.

**Table 5:** Inter- and intra-reader reproducibility of ABC-classification on transesophageal echocardiography at 14, 45, and 90 days

**TEE inter-reader reproducibility**					**TEE intra- reader reproducibility**				
***Time point***	***Agreement***		***Reliability***		***Time point***	***Agreement***		***Reliability***	
14 days	Agreement:	78.69% (42.95%)	Tau-*b*:	0.72	14 days	Agreement	90.32% (43.26%)	Tau-*b*:	0.83
	*Kappa:*	0.63				*Kappa*	0.83		
45 days	Agreement:	72.73% (42.55%)	Tau-*b*:	0.61	45 days	Agreement	86.44% (40.71%)	Tau-*b*:	0.83
	*Kappa:*	0.53				*Kappa*	0.77		
90 days	Agreement:	82.35% (43.02%)	Tau-*b*:	0.75	90 days	Agreement	85.45% (40.69%)	Tau-*b*:	0.81
	*Kappa:*	0.69				*Kappa*	0.75		

*Agreement*, absolute agreement (expected chance agreement); *Kappa*, Cohen’s unweighted kappa statistic. *Tau-*b: Kendall’s tau

### Performance of the ABC classification applied to TEE

**Fig. 3:**
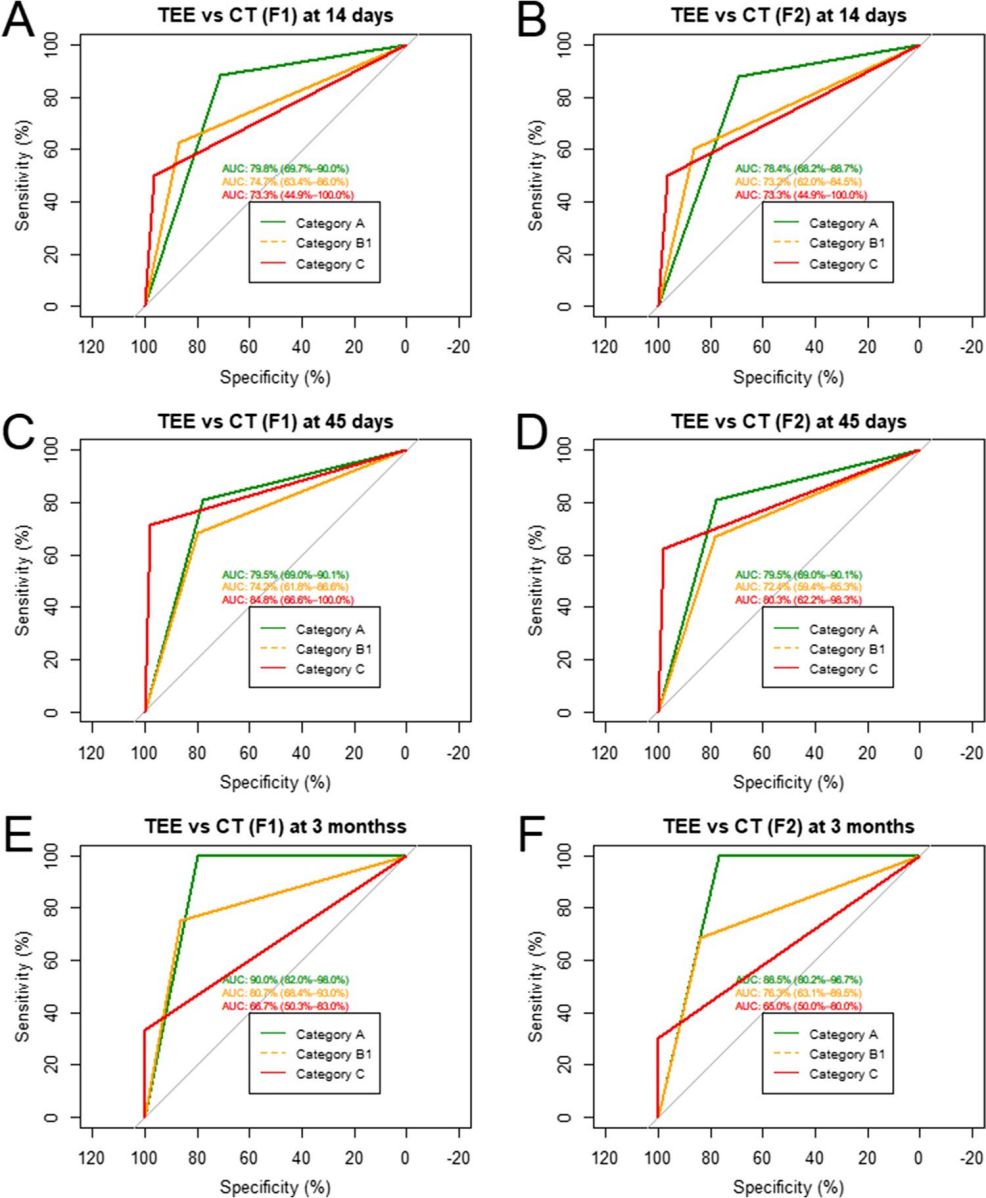
ROC curves of model performance for TEE compared with CT in both flashes and at all 3 timepoints: 14 days (**A**-**B**), 45 days (**C**-**D**), and 90 days (**E**-**F**)

ROC curves for sensitivity and specificity of the ABC-classification applied for TEE versus CCT showed high values of estimated AUC representing good model performance for TEE. The lower AUC values for prediction of Type C were driven by low sensitivity, but with very high specificity, meaning that TEE was reliably able to rule out the high-risk Type C when assigning lower-risk types A and B1 (Fig. 3).

As shown by the Sankey plots (Supplemental Appendix Fig. 1), no discordance from the low-risk type A assigned by TEE to type C on CCT was found in any case.

Type C to type A discordance was very rare and only present in a few 14-day image analyses.

## Discussion

This study tested the feasibility, reliability, and reproducibility of a novel ABC classification for LAA sealing based on the pattern and grade of IDT as evaluated by both CCT and TEE.

The ABC classification was highly reproducible across imaging modalities and readers of differing levels of experience. TEE could reliably classify LAA sealing in agreement with CCT findings. Accordingly, type A patterns detected by TEE were a marker of sealing whereas type C was a marker of patency. Based on frequent transformation of type B patterns into A or C with time, type B patterns can most likely be regarded as small leaks or a transitional phase in device healing.

Cardiac imaging has contributed substantially to the advancement of LAAC [20–22].

Worldwide, image surveillance following LAAC is routinely performed using TEE and/or CCT to examine possible complications such as DRT and PDL as well as to monitor device sealing [23].

The diagnosis of PDL as a suboptimal or unwanted result after LAAC remains a significant challenge [12]. Variabilities in incidence rates and thresholds for clinical significance of leakage possibly stem from differences in definitions, imaging modalities (TEE vs. CCTA), and timing of evaluation [20–22, 24]. TEE remains the least expensive and most versatile imaging modality to support LAAC and is also the most frequently used modality utilized in most RCTs [25]. While offering numerous advantages, the patient compliance for follow-up due to its invasive nature. CCT, on the other hand, is a widely accepted non-invasive alternative to TEE for post-LAAC surveillance and displays superior sensitivity in detecting PDL [15, 17, 26]. Moreover, LAA patency without visible leaks might only be detected by CCT [15, 17, 25].

Despite all advancements in imaging techniques, a substantial discrepancy in leak quantification remains between TEE and CCT. Therefore, the quest for a classification of device sealing status following LAAC, valid across these two dominant imaging modalities, is desirable since a congruent communication between the two primary imaging modalities could help resolve some of the aforementioned remaining uncertainties in PDL evaluation.

Identifying patterns of IDT could facilitate the development of a unified classification system. These patterns may provide a surrogate marker for the process of device healing and even provide clues for the mechanisms of patency. Previous anecdotal studies have reported the feasibility of this approach, but verification of diagnostic accuracy with CCT as well as its reproducibility was lacking [18, 27, 28].

In this cohort, categorizing IDT into three major groups, we showed that TEE was very highly sensitive to detection of almost complete thrombosis of both the device and the LAA (Type A) using CT as the reference. Among patients with Type A, only 2.9% had concurrent CCT-detected PDL at 90 days, mostly of minor size, while no PDLs were found in this group on TEE. On the other hand, Type C patients were associated with PDL in 100% of cases irrespective of imaging modality. TEE showed less sensitivity in detecting Type C patients but with almost perfect specificity. Lower sensitivity might in part be due to innate limitations of the ultrasound itself. Whether using ultrasound-enhancing agents (UEA) might help increase the sensitivity remains to be systematically evaluated in future studies [23].

Considering the geometric characteristics of PDL, it becomes evident that a two-dimensional color-Doppler assessment of the jet may be inadequate for evaluating a crescent-shaped three-dimensional leakage. One recently published retrospective study has attempted to address the problems of 2D quantification of color jets by employing a 3D approach yielding promising results [29]. However inherent limitations of color 3D imaging still restrict its widespread clinical adoption and consistent real-world use.

Clinically significant PDL could also theoretically be caused by multiple gaps of smaller sizes, which further complicate the 2D evaluation of the jet. A non-Doppler-based evaluation of sealing using IDT might help avoid these pitfalls and enhance its clinical reproducibility.

We observed a tendency for larger PDL sizes in patients with Type C at 90 days compared with Type B1. This should be investigated further in a larger study.

The Type B2 represented a rare form of device patency in which failure of IDT was mainly proximal without visible leaks. This might represent a lack of complete device membrane endothelialization. This type was found only during early follow-up imaging (until 45 days) and might imply a transitional stage in the process of device thrombosis and surface healing.

Using a single antiplatelet discharge regimen, the observed DRT rate (4.6%) is aligned with previously observed frequencies [7, 30–33]. Any association between the ABC classification and DRT remains to be further studied.

The substantial inter-reader agreement (kappa range, 0.53–0.69) and intra-reader agreement (kappa range, 0.75–0.83 for TEE to predict the CT-based classification) also confirm the feasibility of this “common language” being implemented regardless of the imaging modality.

We have demonstrated that even with minimal training provided to a single novice reader, consistently high levels of both inter- and intra-reader agreement and reliability were observed across both groups. This underscores the easy applicability of the ABC Classification.

## Clinical perspective

The imaging cutoff for clinically relevant PDL remains a moving target. Using the degree of IDT by TEE and CCT as a surrogate marker of sealing and patency, irrespective of quantitative PDL evaluation, might be useful in initial screening of post-implantation images and might provide clinicians with a baseline for further sophisticated measurements. Despite only minimal training of novice readers in both modalities, we observed good inter-reader and intra-reader reproducibility, indicating that this categorization was robust, feasible, and easy to implement. An approach to the evaluation of LAA sealing and patency based on IDT patterns may improve post-LAAC risk stratification. More research is required to verify the link between IDT and LAA sealing, as well as an association with clinical outcomes.

## Limitations

We acknowledge several limitations in our study. First, this was a single-center observational cohort study with a relatively small sample size, giving rise to inherent limitations such as possible selection bias and lack of generalizability. However, based on the reported baseline characteristics, we do consider our study population comparable with populations described in larger studies. Second, the analysis was based on data obtained from “plug” type devices. The classification was not evaluated for “pacifier” type devices. Third, the clinical outcome of each classification type was not evaluated. To evaluate the associations between IDT patterns and clinical outcomes, this classification needs to be systematically applied to a larger population for sufficient statistical power. Fourth, although inter- and intra-reader agreement was high for CCT and substantial for TEE, image interpretation remains partly subjective and may be influenced by image quality and reader experience. Variability in imaging protocols, scanner settings, or patient-specific anatomical factors may also impact the reproducibility of the classification in external settings.

## Conclusion

The classification of device sealing based on IDT patterns was significantly correlated with the concomitant presence or absence of PDL. It was highly reproducible among readers with varying levels of experience and demonstrated strong diagnostic accuracy across standard cardiac imaging modalities. Type A was associated with sealing regardless of the imaging modality, while Type C was consistently indicative of PDL. Further research utilizing this classification in larger studies is warranted to explore potential correlations with clinical outcomes, which may provide insights into the role of IDT in device healing following LAAC.

## Impact on daily practice

Following LAAC, there is a discrepancy in reported leak quantification between CCT and TEE. In this single-center observational analysis of 68 patients undergoing LAAC with Watchman FLX or FLX-Pro with follow-up by both CCT and TEE at 14, 45, and 90 days, we developed and tested a novel unifying imaging classification based on the patterns and degree of intra-device thrombosis (types A, B, and C). This classification showed high reproducibility and accuracy for prediction of LAA sealing and patency by both CCT and TEE. This preliminary proof-of-concept study requires further validation and research to evaluate the impact of the ABC classification on clinical outcomes.

## Supplementary Information

Below is the link to the electronic supplementary material.

ESM 1 (DOCX 42.6 KB)

## Abbreviations

AF: Atrial fibrillation
CCT: Cardiac computed tomography
DRT: Device-related thrombosis
IDT: Intra-device thrombosis
LAA: Left atrial appendage
LAAC: Left atrial appendage closure
PDL: Peri-device leak
TEE: Transesophageal echocardiography
