# Supplementary material for: The ABC of sealing following left atrial appendage closure
Source: Clin Res Cardiol. 2026 Apr 13;115(6):1016–26. doi: 10.1007/s00392-026-02895-6 (PMC13160979; doi:10.1007/s00392-026-02895-6)
Supplement: Supplementary file 1 — (DOCX 42.6 KB) [file 392_2026_2895_MOESM1_ESM.docx]

**SUPPLEMENTAL APPENDIX**

| Title | Page |
| --- | --- |
| Figure S1  Sankey plots for A) TEE vs CT (F1) at 14 days, B) TEE vs CT (F2) at 14 days, C) TEE vs CT (F1) at 45 days, D) TEE vs CT (F2) at 45 days, E) TEE vs CT (F1) at 90 days, F) TEE vs CT (F2) at 90 days | Pg. 10 |
| Table S1  Quality assessment and interpretation of Cohen’s kappa statistics. | Pg. 10 |
| Table S2  Sensitivity and specificity of TEE in comparison to CCT findings | Pg.10 |
| Table S3  **Intra -reader reproducibility of ABC-classification on cardiac CT at 14, 45, and 90 days on Flash 1 (0 seconds) and Flash 2 (4 seconds)**. | Pg.10 |
|  |  |

**Table S1 :** Quality assessment and interpretation of Cohen’s kappa statistics.

**Table S2:**  Sensitivity and specificity of TEE in comparison to CCT findings (both flashes, 14 days, 45 days and 90 days). The mean overall sensitivity and specificity were obtained by averaging sensitivity and specificity of all categories respectively.

| Modalities and Timing | Diagnostic tests performance | Overall (Average) | Class: A | Class: B | Class: C |
| --- | --- | --- | --- | --- | --- |
| 1. TEE vs CT (F1) at 14 days | Sensitivity | 0.669 | 0.882 | 0.625 | 0.500 |
| - Accuracy 0.758 (CI: 0.633, 0.858) | Specificity | 0.849 | 0.714 | 0.868 | 0.965 |
| 1. TEE vs CT (F2) 14 days | Sensitivity | 0.660 | 0.879 | 0.600 | 0.500 |
| - Accuracy 0.742 (CI: 0.615, 0.845) | Specificity | 0.840 | 0.690 | 0.865 | 0.966 |
| 1. TEE vs CT (F1) at 45 days | Sensitivity | 0.726 | 0.813 | 0.650 | 0.714 |
| - Accuracy 0.746 (CI: 0.616, 0.850) | Specificity | 0.851 | 0.778 | 0.795 | 0.981 |
| 1. TEE vs CT (F2) 45 days | Sensitivity | 0.690 | 0.813 | 0.632 | 0.625 |
| - Accuracy 0.729 (CI: 0.597, 0.836) | Specificity | 0.844 | 0.778 | 0.775 | 0.980 |
| 1. TEE vs CT (F1) at 90 days | Sensitivity | 0.6944 | 1.000 | 0.750 | 0.333 |
| - Accuracy 0.811 (CI: 0.680, 0.906) | Specificity | 0.8883 | 0.849 | 0.706 | 1.000 |
| 1. TEE vs CT (F2) at 90 days | Sensitivity | 0.6625 | 1.000 | 0.6875 | 0.300 |
| - Accuracy 0.774 (CI: 0.6379, 0.8772) | Specificity | 0.8690 | 0.769 | 0.838 | 1.000 |

**Table S3 : Intra -reader reproducibility of ABC-classification on cardiac CT at 14, 45, and 90 days on Flash 1 (0 seconds) and Flash 2 (4 seconds)**. Agreement: Absolute agreement (expected chance agreement), Kappa: Cohen’s unweighted kappa statistic. Tau-b: Kendall’s tau.

| CT intra- reader reproducibility (reader 1) | | | | | CT intra- reader reproducibility (reader 2) | | | | |
| --- | --- | --- | --- | --- | --- | --- | --- | --- | --- |
| *Time and flash* | ***Agreement*** | | ***Reliability*** | | ***Time and flash*** | ***Agreement*** | | ***Reliability*** | |
| 14 days F1 | Agreement: | 95.00% (42.97%) | Tau-*b*: | 0.92 | **14 days F1** | Agreement | 100.0% (43.91%) | Tau-*b*: | 1.00 |
|  | *Kappa:* | 0.91 |  |  |  | *Kappa* | 1.00 |  |  |
| 14 days F2 | Agreement: | 98.33% (42.69%) | Tau-*b*: | 0.97 | **14 days F2** | Agreement | 98.31% (48.69%) | Tau-*b*: | 0.97 |
|  | *Kappa:* | 0.97 |  |  |  | *Kappa* | 0.97 |  |  |
| 45 days F1 | Agreement: | 94.74% (37.06%) | Tau-*b*: | 0.94 | **45 days F1** | Agreement | 93.65% (39.83%) | Tau-*b*: | 0.92 |
|  | *Kappa:* | 0.92 |  |  |  | *Kappa* | 0.89 |  |  |
| 45 days F2 | Agreement: | 91.23% (35.92%) | Tau-*b*: | 0.90 | **45 days F2** | Agreement | 95.24% (37.14%) | Tau-*b*: | 0.95 |
|  | *Kappa:* | 0.86 |  |  |  | *Kappa* | 0.92 |  |  |
| 90 days F1 | Agreement: | 96.55% (38.02%) | Tau-*b*: | 0.97 | **90 days F1** | Agreement | 90.32% (40.48%) | Tau-*b*: | 0.87 |
|  | *Kappa:* | 0.94 |  |  |  | *Kappa* | 0.84 |  |  |
| 90 days F2 | Agreement: | 93.22% (37.89%) | Tau-*b*: | 0.93 | **90 days F2** | Agreement | 87.10% (38.81%) | Tau-*b*: | 0.80 |
|  | *Kappa:* | 0.92 |  |  |  | *Kappa* | 0.79 |  |  |

**Figure S1**: Sankey plots for A) TEE vs CT (F1) at 14 days, B) TEE vs CT (F2) at 14 days, C) TEE vs CT (F1) at 45 days, D) TEE vs CT (F2) at 45 days, E) TEE vs CT (F1) at 90 days, F) TEE vs CT (F2) at 90 days (cases with missing values were excluded in each pairwise comparison).

**
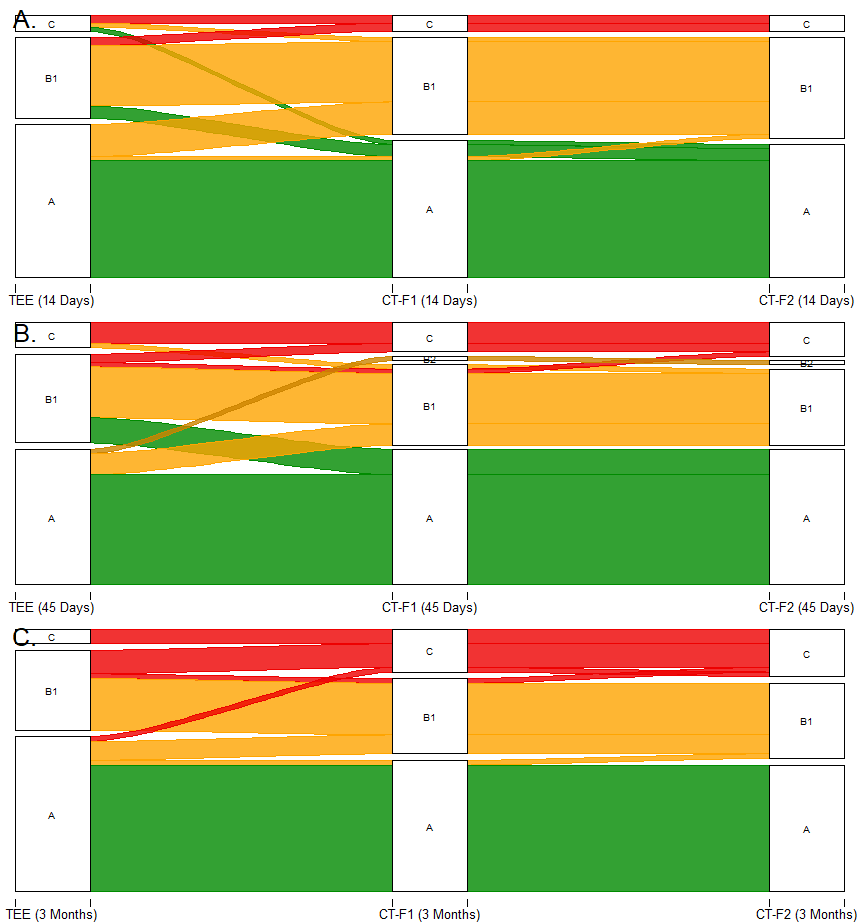
**
